# Advances in the Use of Recombinant Gonadotropins for Reproductive Biotechnologies in Ruminants: A Review

**DOI:** 10.1111/rda.70216

**Published:** 2026-05-14

**Authors:** Vitória Leite Di Domenico, Fabiane Pereira de Moraes, Natália Ávila de Castro, Fernando Caetano de Oliveira, Diogo Magnabosco, Bernardo Garziera Gasperin

**Affiliations:** ^1^ Programa de Pós‐Graduação em Zootecnia UFRGS Porto Alegre Brazil; ^2^ Programa de Pós‐Graduação em Veterinária UFPel Pelotas Brazil; ^3^ Faculdade de Veterinária UFRGS Porto Alegre Brazil

**Keywords:** animal welfare, reproductive biotechnology, timed artificial insemination

## Abstract

Recombinant gonadotropins, particularly recombinant follicle‐stimulating hormone (rFSH) and recombinant equine chorionic gonadotropin (reCG), have emerged as viable alternatives to traditional animal‐derived hormones in reproductive biotechnologies. This review aims to provide an updated and comprehensive overview of recombinant gonadotropins, with emphasis on the most recent scientific and technological advances in production and their application. This research integrates studies published following the recent commercial introduction of these molecules and incorporates findings generated by our research group. A total of 12 field studies in ruminants met the inclusion criteria, comprising four evaluating rFSH for superovulation and eight assessing reCG across different reproductive applications. Recent studies demonstrate that rFSH achieves similar superovulatory outcomes to conventional protocols, while reCG effectively promotes follicular growth, ovulation, and fertility in cattle and sheep, with results comparable to purified eCG. Beyond reproductive efficacy, recombinant hormones offer advantages such as reduced animal use, consistent product quality, and simplified management. These findings highlight recombinant gonadotropins as promising, ethical, and efficient tools for advancing assisted reproduction in ruminants.

## 1 Introduction

Gonadotropins, including follicle‐stimulating hormone (FSH) and luteinizing hormone (LH), are glycoproteic hormones primarily synthesized and secreted by the anterior pituitary gland in response to gonadotropin‐releasing hormone (GnRH) synthesized and secreted by the hypothalamus (De Koning et al. 1994). FSH stimulates granulosa cells, promoting follicular recruitment and growth (Gifre et al. 2017), whereas LH is involved in final follicular growth and is essential for ovulation of the pre‐ovulatory follicle. In certain species, gonadotropins can also be produced by the placenta, such as equine chorionic gonadotropin (eCG) (Hassanein et al. 2024), a glycoproteic hormone that, in non‐equine species, exhibits follicle‐stimulating and luteinizing activity (Stewart and Allen 1981).

Most gonadotropins used for reproductive purposes are obtained from animal‐derived biological sources, requiring extraction and purification (Baruselli et al. 2023; Gifre et al. 2017). However, purified hormones tend to present batch‐to‐batch variation, resulting from the inherent complexity of biological extraction and purification processes, including the presence of contaminating hormones and other proteins (Lösle et al. 2025). Furthermore, the production of eCG raises significant animal welfare concerns, as it relies on repeated blood collections from pregnant mares (reviewed by Baruselli et al. 2023).

Recombinant biotechnology has enabled the production of a wide range of proteins, including FSH and eCG, using mammalian and fungal expression systems (Gifre et al. 2017). The advantages of recombinant hormones are greater consistency and reproducibility, no need for extraction and purification from animals, reduced risk of pathogen transmission, and enhanced quality control (Baruselli et al. 2023; Villarraza et al. 2021). Additionally, according to a review by Baruselli et al. (2023), recombinant gonadotropins can be engineered to exhibit prolonged biological activity, enabling single‐dose administration, reducing handling.

The use of recombinant hormones in animal reproduction represents a promising alternative to conventional approaches. Therefore, this review aims to provide an updated and comprehensive overview of recombinant gonadotropins, with emphasis on the most recent scientific and technological advances, integrating studies published following the recent commercial introduction of these molecules and incorporating findings generated by our research group. Unlike previous reviews, which primarily focused on conventional gonadotropins or early experimental developments, this article synthesizes recent datasets from in vivo applications across different reproductive biotechnologies. Furthermore, this review discusses data from the use of recombinant and conventional molecules under commercial conditions, thereby highlighting current gaps and identifying directions for future research and application.

## 2 Recent Advances in Recombinant FSH Development

Ovarian hyperstimulation currently relies on the administration of pituitary‐derived FSH, in combination with other hormones, to promote the growth and maturation of multiple follicles and thereby achieve superovulation (Abreu et al. 2024). However, purified FSH (pFSH) is characterized by a short half‐life of approximately 5 h in cows, due to rapid metabolic clearance and distribution to peripheral tissues (Bó and Mapletoft 2020; Laster 1972). Therefore, purified FSH treatment requires multiple daily administrations to sustain effective follicular stimulation.

Due to the limitations associated with the use of purified gonadotropins, recent studies have explored biotechnological alternatives to produce recombinant FSH (rFSH). The recombinant human FSH (rhFSH; follitropin alfa) is a gonadotropin of human origin produced in Chinese hamster ovary (CHO) cells (Khodadadi et al. 2022), which is an immortalized cell line. CHO represents the primary mammalian expression system for large‐scale production of recombinant proteins, owing to the ability to perform efficient post‐translational modifications comparable to those of human cells (Li et al. 2022). rhFSH application in ruminant reproduction has been explored in superovulation for in vivo or in vitro embryo production. However, as noted by Mirzaei et al. (2024), the repeated use of a molecule of human origin may trigger an immunogenic response, potentially reducing the efficacy of rhFSH and long‐acting (LA)‐rhFSH.

Considering the immunogenicity‐related issues, there has been a demand to produce specific recombinant gonadotropins for veterinary use. Cabeza et al. (2024), using CHO cell culture, developed and characterized a novel single‐chain bovine FSH variant (bscrFSH—bovine ripafollitropin alfa) designed to overcome the limitations of pituitary‐derived porcine FSH, such as LH contamination, the potential to trigger an immune response in cattle, and the short half‐life. To prolong its half‐life, the molecule was engineered to contain additional N‐glycosylation sites to increase its molecular weight. Superovulation trials in cattle in a decreasing four‐dose regimen confirmed the molecule's efficacy, comparable to outcomes achieved with pituitary‐derived pFSH.

**FIGURE 1:**
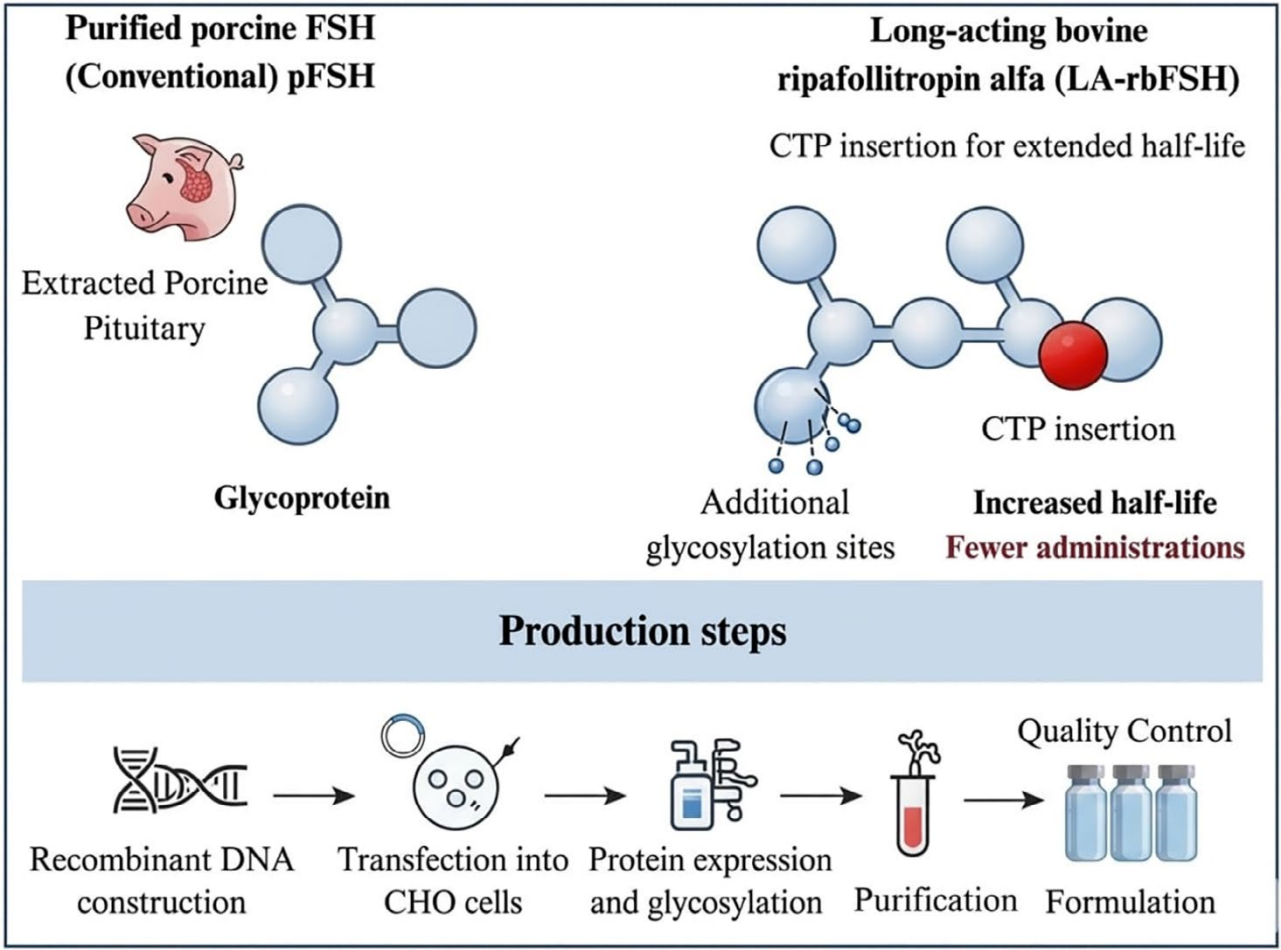
Schematic diagram comparing the glycoprotein structures of conventional purified porcine FSH (extracted from porcine pituitary gland) and long‐acting recombinant bovine ripafollitropin alfa. Arrows highlight key recombinant modifications in the long‐acting version, including additional glycosylation sites and C‐terminal peptide (CTP) insertion, which confer extended half‐life and reduced administration frequency. The integrated flowchart depicts the recombinant production process: (1) recombinant DNA construction (with CTP modification), (2) transfection into CHO cells, (3) protein expression and glycosylation, and (4) purification. CHO: Chinese hamster ovary; CTP: C‐terminal peptide; FSH: follicle‐stimulating hormone.

The search for alternatives to reduce the number of FSH administrations in bovine superovulation protocols has led to the development of long‐acting molecules such as human (LA‐rhFSH; human corifollitropin alfa) and bovine FSH (LA‐rbFSH; bovine ripafollitropin alfa). The long‐acting molecules are engineered to prolong their half‐life through the insertion of a carboxy‐terminal peptide (CTP) containing multiple glycosylation sites (Ben‐Menahem 2018) (Figure 1). Recent studies in cattle suggest that LA‐rhFSH can promote sustained follicular growth and simplify superovulation protocols with only one FSH application, thereby reducing animal handling, labour, and costs (Viana et al. 2024; Mirzaei et al. 2024). However, as rhFSH, its human origin may cause immunogenic reactions that compromise the results of the protocol.

Accordingly, due to the demand for molecules of veterinary origin, Villarraza et al. (2024) developed a LA‐rbFSH produced in CHO. The LA‐rbFSH variant exhibited a purity level of 99%, with a more extensive glycosylation profile and higher sialic acid content—characteristics associated with enhanced circulatory stability. Furthermore, LA‐rbFSH demonstrated higher plasma concentrations and a specific in vivo bioactivity 2.4 times greater than bscrFSH.

Abreu et al. (2024) investigated an alternative to CHO, the non‐pathogenic unicellular protozoan *Leishmania tarentolae* as a host to produce bioactive bscrFSH. *L. tarentolae* can be cultured in vitro at high cell densities and exhibits a glycosylation highly similar to mammalian N‐glycosylation. They produced a biologically active form of bscrFSH, which stimulated follicular growth in a dose‐dependent manner. This study represents an advance in the use of non‐conventional systems for the expression of complex glycoprotein hormones, highlighting a growing trend toward reducing production costs and avoiding the use of serum or animal‐derived cell lines. However, optimization of the expression system to enhance molecular stability and in vivo half‐life and dose–response studies in ruminants are necessary.

Although a few molecular modification strategies have yielded promising results in terms of stability, half‐life, and biological performance, to date, the only commercially available bovine recombinant FSH for use in livestock in South America is a ready‐to‐use long‐acting injectable formulation containing LA‐rbFSH (Zimbria, Ceva Saúde Animal). LA‐rbFSH is indicated for use in pubertal and cyclic female cattle in superovulation (SOV) and embryo transfer (ET) protocols, administered as a single dose. This molecule combines prolonged half‐life properties with effective biological activity and is currently employed in reproductive management protocols.

### 2.1 Studies Using Bovine Recombinant FSH in Ruminants

Given the growing interest in recombinant FSH molecules, studies have compared their effectiveness with purified FSH sources. Gutierrez‐Reinoso et al. (2025) evaluated bscrFSH in new SOV protocols for lactating dairy cows, comparing four applications (every 24 h) with two applications (at 48 h intervals) of 240 μg total dose. Cows receiving four injections showed more ovarian structures on day 8 (14.4 ± 1.2 vs. 12.8 ± 0.9), more transferable embryos (6.0 ± 0.5 vs. 5.2 ± 0.2), and fewer unfertilized oocytes (0.1 ± 0.0 vs. 0.3 ± 0.1). Despite these differences, circulating concentrations of 17β‐estradiol, FSH, LH, and P4 were not affected by treatments. These results indicate that the four‐application bscrFSH protocol is effective while requiring less handling than conventional regimens involving six to eight injections of purified FSH, and that the two‐application protocol can also induce a satisfactory superovulatory response, further reducing costs and handlings.

Based on the advances achieved with bscrFSH, new sustained‐release formulations are being developed to reduce the number of injections and simplify management during superovulation in ruminants. In a recent study conducted by our group, Frata et al. (2025) compared conventional pFSH (225–450 IU) administered in eight doses with a single application of LA‐rbFSH (125–200 μg) for superovulation in *Bos taurus* and crossbreed cows and heifers and found no differences in total structures or transferable embryos. However, LA‐rbFSH resulted in a higher proportion of unfertilized oocytes (4.5% vs. 0%), whereas pFSH resulted in more degenerated embryos (25% vs. 18.4%). Furthermore, it is important to highlight that repeating the LA‐rbFSH treatment in the same cows up to four times did not affect the superovulatory response or the number of embryos. Da Silva et al. (2025) reported that administering 100 μg LA‐rbFSH on day 4 of a synchronization protocol increased embryo production (5.0 vs. 2.8) and blastocyst rate (40.6% vs. 31.1%) compared with the control group in Gir cows ( *Bos indicus* ) undergoing oocyte retrieval for in vitro fertilization. Therefore, these results indicate that LA‐rbFSH is not only as effective as the conventional pFSH protocol but also reduces animal handling.

When evaluating the effects of follicular synchronization with recombinant bovine somatotropin (rBST) or LA‐rbFSH on follicular distribution, germinal vesicle (GV) stages, and transzonal projection (TZP) density in Nelore heifers and cows undergoing oocyte retrieval by OPU, Perecin et al. (2025) observed that, in heifers, both the control and rBST protocols yielded more COCs from small follicles, whereas LA‐rbFSH increased the recovery of oocytes from large follicles and the proportion at the GV3 stage. In cows, rBST also promoted the retrieval of COCs from small follicles compared with the control and LA‐rbFSH groups; however, no significant differences were found in GV‐stage distribution or TZP density. These results demonstrate that recombinant hormones can be strategically selected to modulate GV‐stage enrichment and improve in vitro maturation protocols.

These findings highlight the potential of recombinant gonadotropins, particularly bscrFSH and LA‐rbFSH as promising alternatives to porcine pituitary‐derived products. Additionally, they offer the advantage of being developed based on the bovine FSH sequence, in contrast to rhFSH and LA‐rhFSH (Viana et al. 2024; Khodadadi et al. 2022). Although some limitations are still observed, including the need for optimization of dose and administration protocols and limited availability of large‐scale field studies, recombinant FSH has shown comparable or even superior biological performance across different production contexts (Table 1), while offering notable advantages such as the feasibility of single‐dose administration and, consequently, fewer handling procedures, thereby minimizing animal stress. Moreover, advances in cellular biotechnology, especially using CHO cell lines, have enabled the large‐scale production of these molecules with improved standardization and potential cost reductions in the medium and long term. Therefore, the use of rFSH is a promising tool for developing more efficient, standardized, and sustainable assisted reproduction protocols, particularly in response to the growing demand for safe and reproducible reproductive biotechnologies with minimal impact on animal management.

**TABLE 1:** Main ovarian and embryo production outcomes of studies evaluating recombinant follicle‐stimulating hormone (rFSH) in ruminants submitted to superovulation protocols.

References	Molecule	Species and category	Dose	Main results
Gutierrez‐Reinoso et al. (2025)	bscrFSH	Lactating dairy cows ( *Bos taurus* )	Four or two applications of 240 μg	Greater number of total structures (14.4 ± 1.2 vs. 12.8 ± 0.9) and transferable embryos (6.0 ± 0.5 vs. 5.2 ± 0.2) for 4 vs. 2 applications, respectively
Frata et al. (2025)	LA‐rbFSH e pFSH	*Bos taurus* and crossbreed cows and heifers	Heifers: 125–150 μg LA‐rbFSH or 225–300 IU pFSH. Cows: 160–200 μg LA‐rbFSH or 300–450 IU pFSH	Total structures and transferable embryos did not differ between pFSH (8 doses) and LA‐rbFSH (single dose) treatments
Da Silva et al. (2025)	LA‐rbFSH	Gir ( *Bos indicus* ) cows	Control or 100 μg LA‐rbFSH	Increased in vitro embryo production (5.0 vs. 2.8) and a higher blastocyst rate (40.6% vs. 31.1%) for LA‐rbFSH compared with the control group
Perecin et al. (2025)	LA‐rbFSH e rBST	Nelore ( *Bos indicus* ) heifers and cows	Control, 100 μg LA‐rbFSH or 325 mg rBST	LA‐rbFSH increased the recovery of large‐follicles COCs and GV3 oocytes, and no differences were observed in GV stages or TZP density among treatments

Abbreviations: μg: microgram; bscrFSH: recombinant bovine single‐chain follicle‐stimulating hormone (bovine ripafolitropin alfa); GV: germinal vesicle; IU: international units; LA‐rbFSH: recombinant bovine long‐acting follicle‐stimulating hormone (bovine ripafolitropin alfa); pFSH: purified follicle‐stimulating hormone; rBST: recombinant bovine somatotropin; TZP: transzonal projections.

## 3 Recent Advances in Recombinant eCG Development

The eCG is a glycoproteic hormone produced by chorionic cells of pregnant mares, and it was first identified in 1930 as a factor present in the blood during the first third of gestation (Cole and Hart 1930). It is secreted by endometrial cups between days 37 and 120 of pregnancy in equine species and, like other chorionic gonadotropins, contains α and β subunits, with a high carbohydrate content, primarily composed of sialic acid residues that confers an extended half‐life (Flores‐Flores et al. 2014). In mares, eCG binds mainly to LH receptors (Stewart and Allen 1981), inducing accessory CLs during pregnancy; however, due to its unique structure, eCG can bind to both FSH and LH receptors in non‐equid species (reviewed by Murphy 2012).

In cattle, the administration of eCG has been associated with beneficial effects on both follicular and luteal development, particularly in females in anestrus and with low body condition scores (BCS) (Baruselli et al. 2004). Depending on the dose and moment of administration, eCG treatment in cows reduces follicular atresia, increases recruitment and growth of small to large follicles, improves dominant and preovulatory follicle development, resulting in better CL quality, greater progesterone secretion, and higher pregnancy rates (De Rensis and López‐Gatius 2014).

Despite its benefits, the commercial use of eCG has several limitations. Currently, the available formulations are extracted and purified from the blood of pregnant mares in early gestation, a process associated with major drawbacks, including animal welfare concerns due to repeated blood collections and, sometimes, pregnancy interruption, as well as marked batch‐to‐batch variability (Villarraza et al. 2021; Vilanova et al. 2019). Lösle et al. (2025) reported only 5.5% of similarity in protein composition when comparing four commercially available purified eCG products, highlighting substantial discrepancies among products on the market.

Given the well‐documented benefits of eCG and the limitations of its purified form, recombinant eCG (reCG) has become a promising alternative, offering controlled production, elimination of animal use, and greater batch consistency. Villarraza et al. (2021) produced reCG in CHO‐K1 cells—a variant of CHO cells—using lentiviral vector systems as the gene delivery method, generating biologically active hormone. Subsequently, Byambaragchaa et al. (2025) also established a reCG system in CHO‐DG44 cells (derived from CHO‐K1) expressing a single‐chain form. The produced reCG activated FSH and LH receptors (eLH/CGR, rLH/CGR, rFSHR), stimulating cAMP production, pERK1/2 signalling, and β‐arrestin 2 recruitment in a dose and time‐dependent manner. These findings confirmed the biological functionality of reCG, capable of activating multiple intracellular pathways, and highlighted its potential as a promising alternative to replace animals as the source of eCG.

In addition to eliminating the dependence on pregnant mares, recombinant production offers greater control over molecular structure, potential for industrial scaling, and long‐term cost reduction. Additional advantages include reduced labour demands by providing a ready‐to‐use formulation, reducing the risk of product loss or dosing errors. Improved standardization of biological responses further offers economic benefits through greater predictability and reduced losses from ineffective treatments. Thus, reCG represents not only a biotechnological innovation but also a sustainable and ethically preferable alternative for livestock reproductive management. Similar to rFSH, a single commercial product is currently available in Brazil (Foli‐Rec, Ceva Saúde Animal), with availability extending to other countries, supplied as a ready‐to‐use injectable solution for follicular growth induction in bovine TAI protocols.

### 3.1 Studies Using reCG in Ruminants

With advances in gene expression and cell culture technologies, several strategies have been developed to produce a functional reCG with biological activity comparable to the native molecule. Dose–response and field studies have been conducted in different animal models (Table 2). Villarraza et al. (2021) evaluated reCG in cows and heifers under field conditions, comparing its performance with purified eCG, and the results varied according to dose and protocol. In anestrous cows, the 140 IU dose achieved the best outcomes, with pregnancy rates of 45%, not differing from eCG (42.9%). In anestrous lactating crossbred beef cows, 140 IU of reCG and 400 IU of eCG produced similar dominant follicle diameters, and both increased ovulation rates relative to controls; however, ovulation occurred earlier with reCG. In another trial with Angus cows (up to 90 days postpartum), the same pregnancy rate (58%) was obtained with 140 IU of reCG or 400 IU of eCG, both significantly higher than other treatments and the control group. Collectively, these findings demonstrate that reCG has the potential to replace eCG in TAI.

**TABLE 2:** Main reproductive outcomes of studies evaluating reCG in ruminants in different reproductive biotechnologies (FTAI, ovulation induction, embryo transfer and superovulation).

References	Animals	Main results
Villarraza et al. (2021)	Crossbred cows	Pregnancy rate was significantly lower with the use of 400 IU reCG compared to 400 IU eCG (12% vs. 58%)
	Anestrous cows	Pregnancy rates after FTAI did not differ among 100 IU reCG (33%), 140 IU reCG (45%), 200 IU reCG (30%) and 400 IU eCG (42.9%)
	Holstein heifers	After SOV, 2000 IU reCG increased the number of follicles ≥ 8 mm, CL number, and follicular perfusion compared with 1000 IU reCG. CL number did not differ between 2000 IU reCG and 2500 IU eCG
	Anestrous lactating crossbred cows	Follicular dynamics during FTAI protocol revealed: no difference for dominant follicles diameter; higher ovulation rate in eCG‐treated groups (140 IU reCG: 80%; 400 IU eCG: 71%) compared to control (41%); and earlier ovulation in reCG and control compared to eCG‐treated cows
	Suckled Angus cows	Higher pregnancy rate in reCG‐treated (reCG: 54% for 105 IU; 57% for 120 IU; and 58% for 140 IU) and 400 IU eCG‐treated (58%) cows compared to untreated control (41%)
Cattaneo et al. (2024)	Suckled beef cows	Higher estrus rate and P/AI after FTAI in reCG‐treated cows (105 IU: 79.9% and 53.5%; 140 IU: 76.9% and 52.3%) compared to control group (69.9% and 44.4%). Higher P/AI after FTAI for reCG (84 IU: 38.6%; 105 IU: 37.1%; 140 IU: 36.2%) compared to untreated control (28.2%). No difference in P/AI between 84 IU reCG (54%) and 300 IU eCG (59%), and lower P/AI in cows treated with 105 IU reCG (41%)
Carvalho et al. (2026)	Dairy cows	Both doses increased P/AI after FTAI (45.2% for 105 IU and 42% for 140 IU reCG) compared to control (34.1%).
Rodríguez et al. (2024)	Heifers, primiparous and multiparous Angus cows	In a Co‐synch FTAI protocol: higher estrus expression after treatment with eCG‐like (400 IU) compared to untreated control in primiparous (68.9% vs. 45.0%) and multiparous (75.5% vs. 68.8%) cows. In heifers 300 IU eCG‐like increased conception rate (65.2%), compared to untreated control (48.3%). The same was observed for primiparous cows treated with 400 IU eCG‐like (48.3% vs. 35.1%); CL area increased in heifers and multiparous cows, and progesterone tended to be higher in heifers treated with eCG‐like
Camozzato et al. (2026)	Anestrous ewes	Higher follicular growth for 105 IU reCG and 400 IU eCG compared to untreated control. Ovulation rate was higher for reCG (88.9%) and eCG (100%) compared to control (0%). Serum P4 was lower for 105 IU reCG compared to 400 IU eCG; pregnancy and conception rates during seasonal transition were not affected by eCG treatments
Rodrigues et al. (2025)	Crossbred cows	No difference in the percentage of treated‐to‐transferred rate (eCG 300 IU: 93.0%; reCG 105 IU: 85.0% and reCG140 IU: 84.4%) and in P/ET (eCG 300 IU: 42.3%; reCG 105 IU: 39.3%; and reCG140IU: 44.6%). Pregnancy loss was not affected
Gretter et al. (2025)	Nellore cows	After SOV, similar ovulation rate and embryo viability were observed among treatments with 100 μg LA‐bscrFSH, 100 μg LA‐bscrFSH + 175 IU reCG or 1.050 IU reCG; lower recovery rate with reCG alone; the rFSH + reCG combination did not improve embryo production, and reCG was as effective as rFSH
Bandeo et al. (2025)	Buffaloes	Higher‐quality oocytes obtained after treatment with 160 mg FSH and 2500 IU eCG. Embryo production was higher in buffaloes treated with FSH, but did not differ from 1050 IU reCG (1.5 vs. 1.05 embryos/buffalo/OPU). Higher cleavage rates in reCG‐treated

Abbreviations: eCG: equine chorionic gonadotropin; eCG‐like: eCG‐like glycoprotein; FTAI: fixed‐time artificial insemination; FTET: fixed‐time embryo transfer; IU: international units; LA‐bscrFSH: long‐acting bovine recombinant follicle‐stimulating hormone (bovine ripa‐follitropin alpha); P4: progesterone; reCG: recombinant equine chorionic gonadotropin.

Continuing the evaluation of reCG under different production conditions, Cattaneo et al. (2024) assessed its effects on pregnancy rates in lactating beef cows synchronized with an estradiol/progesterone‐based TAI protocol. When comparing 140 and 105 IU, cows treated with reCG showed higher estrus expression and pregnancy per artificial insemination (P/AI) (105 IU: 79.9% and 53.5%; 140 IU: 76.9% and 52.3%) compared with control cows (69.9% and 44.4%). In subsequent trial testing 140, 105, and 84 IU, all reCG doses tended to increase P/AI relative to control (84 IU: 38.6%; 105 IU: 37.1%; 140 IU: 36.2%; control: 28.2%). Furthermore, reCG was compared with 300 IU of eCG. Cows receiving 84 IU of reCG achieved a P/AI (54%) similar to eCG (59%), both outperforming the 105 IU reCG group (41%). Overall, the authors concluded that reCG enhances fertility in suckling beef cows and that, although its efficacy is comparable to eCG, excessive reCG doses may reduce P/AI.

Building upon this evidence, subsequent studies have further explored the influence of reCG dosage and protocol duration on fertility outcomes in cattle. In Holstein and crossbreed dairy cows, Carvalho et al. (2026) conducted two experiments evaluating the efficacy of reCG. In the first trial, greater pregnancy rates were obtained after TAI in Holstein cows treated with 400 IU eCG (36.8%) or 140 IU reCG (37.2%), compared to untreated cows (27.8%). In the second experiment, no significant difference was observed in pregnancy rates after TAI in Holstein and Holstein × Gir cows treated with 105 IU reCG (45.2%) or 140 IU reCG (42%), both treatments being superior to the control (untreated) group (34.1%). Together with previous results, these data reinforce the equivalence between eCG and reCG.

Expanding this perspective, Rodríguez et al. (2024) demonstrated that the administration of an eCG‐like glycoprotein improves reproductive performance in cattle. In Angus cows synchronized with a 5‐day Co‐Synch protocol, the treatment increased ovulation and TAI pregnancy rates in primiparous (68.9% vs. 45.0%) and multiparous (75.5% vs. 68.8%) cows compared with controls. Conception rates also increased in heifers (65.2% vs. 48.3%), primiparous cows (48.3% vs. 35.1%), and multiparous cows with a BCS ≤ 4 (47.7% vs. 34.8%). Moreover, the eCG‐like treatment increased CL area in heifers and multiparous cows and tended to elevate serum progesterone concentrations in heifers. These results highlight the effectiveness of gonadotropin analogues in enhancing AI programs and the importance of considering category and BCS to optimize reproductive responses.

In a recent study conducted by our group with seasonally anestrous ewes in Southern Brazil, Camozzato et al. (2026) demonstrated that ewes treated with eCG (400 IU) or reCG (105 IU) showed greater follicular growth than controls, with ovulation rates of 100% and 88.9%, respectively, while no ovulations occurred in controls. The CL area and blood perfusion were similar between eCG and reCG treated ewes, whereas serum progesterone concentrations were significantly higher in the eCG group. In a second experiment, estrus expression increased in eCG (95.27%) and reCG (88.97%) groups, compared to control (78.22%), but pregnancy (46.53% to 50%) and conception (51.45% to 56.78%) rates after natural mating did not differ among treatments. These findings indicate that reCG can achieve physiological responses comparable to eCG even during seasonal anestrus; however, further studies are required to evaluate their impact on fertility under deep anestrus conditions.

In addition to TAI applications, recent studies highlight the effectiveness of reCG in SOV and ET. Rodrigues et al. (2025) reported similar ovulation and conception rates when comparing reCG (105 or 140 IU) with purified eCG (300 IU) in ET recipients (47%, 55.1% and 46.8%, respectively), with a tendency for higher responses in primiparous Nelore cows. Regarding in vivo embryo production, in Holstein heifers, 2000 IU of reCG increased the number of large follicles and CL during superovulation compared with 1000 IU reCG. However, there was no significant difference in the number of CL induced by 2000 IU reCG and 2500 IU eCG (Villarraza et al. 2021). Gretter et al. (2025) evaluated rFSH (100 μg rFSH), reCG (1050 UI reCG), and their combination (100 μg rFSH + 175 UI reCG) in SOV protocols for in vivo embryo production in Nelore donors and found similar ovulation rates (rFSH: 59%; rFSH + reCG: 56%; reCG: 56%) and numbers of large follicles and CLs across treatments. The number of viable embryos (rFSH: 4.3; rFSH + reCG: 4.1; reCG: 3.9) and embryonic viability were comparable among groups, indicating that reCG is effective in producing embryos comparable to those obtained with rFSH. The combination of rFSH and reCG did not improve embryo yield, reinforcing that individual use of either gonadotropin can achieve comparable embryonic outcomes. Overall, these findings indicate that reCG is a viable alternative to conventional eCG and FSH in ET and SOV programs, though broader evaluation is still required for its optimized application.

Complementing the findings observed in cattle and sheep, Bandeo et al. (2025) compared pFSH, reCG, and eCG treatments before ovum pick‐up (OPU) in buffaloes and found no differences in the total numbers of follicles, oocytes, or zygotes. Higher‐quality oocytes were obtained with pFSH and eCG, while embryo yield was greater in buffaloes treated with pFSH but did not differ significantly from those treated with reCG (1.5 vs. 1.05 embryos/buffalo/OPU). Cleavage rates, however, were superior after reCG treatment.

The overall evidence indicates that eCG and reCG play a fundamental role in the induction of follicular growth and ovulation, contributing to the success of reproductive programs. The consistent performance of reCG across different species and protocols highlights its biological efficiency and applicability. Continued research is essential to refine dosage and evaluate long‐term fertility outcomes.

## 4 Conclusion

Although the commercial use of recombinant hormones, such as rFSH and reCG, is still in the consolidation phase, studies have demonstrated several advantages. For rFSH, a single administration has proven to be as effective as conventional multi‐dose pFSH for superovulation, while reCG has shown comparable efficacy in promoting follicular growth and ovulation in ruminants. The potential to reduce interventions and handling procedures, decrease labour requirements, increase the predictability of hormonal responses, and eliminate dependence on animal‐derived products underscores their value as a biotechnological tool aligned with the demands for production efficiency, animal welfare and biosecurity.

Although recombinant hormones still have a high initial cost and, at certain doses, may be more expensive than conventional purified hormones, their potential for cost reduction over time is noteworthy. As production scale increases, the cost per unit tends to decrease, making these products progressively more competitive. In addition, standardized production, greater hormone availability, and a high degree of purity may improve use efficiency, helping to reduce the cost per dose and expand their applicability.

Further studies should be developed to validate the use of recombinant gonadotropins in other species, as well as to consolidate their use in cattle and sheep to refine indices and strategies.

## Author Contributions

Vitória Leite Di Domenico, Fabiane Pereira de Moraes and Natália Ávila de Castro conducted the literature review and the initial draft of the manuscript; Fernando Caetano de Oliveira and Diogo Magnabosco provided overall supervision and co‐edited the manuscript; Fernando Caetano de Oliveira and Bernardo Garziera Gasperin edited and reviewed the final manuscript.

## Conflicts of Interest

The authors declare no conflicts of interest.

## Data Availability

Data sharing not applicable to this article as no datasets were generated or analysed during the current study.

## References

[rda70216-bib-0001] Abreu, C. , K. Grunberg , M. Bonilla , et al. 2024. “Expression and Functional Characterization of Chimeric Recombinant Bovine Follicle‐Stimulating Hormone Produced in Leishmania Tarentolae.” Microbial Biotechnology 17, no. 4: e14444. 10.1111/1751-7915.14444. 38564168 PMC10986757

[rda70216-bib-0002] Bandeo, A. , J. L. Konrad , P. Ponce , et al. 2025. “In Vitro Production and Transfer of Buffalo Embryos ( *Bubalus bubalis* ) in Argentina.” Journal of Buffalo Science 14: 20–28. 10.6000/1927-520X.2025.14.03.

[rda70216-bib-0003] Baruselli, P. S. , L. Â. de Abreu , B. L. C. Catussi , et al. 2023. “Use of New Recombinant Proteins for Ovarian Stimulation in Ruminants.” Animal Reproduction 20, no. 2: e20230092. 10.1590/1984-3143-AR2023-0092. 37720727 PMC10503889

[rda70216-bib-0004] Baruselli, P. S. , E. L. Reis , M. O. Marques , L. F. Nasser , and G. A. Bó . 2004. “The Use of Hormonal Treatments to Improve Reproductive Performance of Anestrous Beef Cattle in Tropical Climates.” Animal Reproduction Science 82–83: 479–486. 10.1016/j.anireprosci.2004.04.025. 15271474

[rda70216-bib-0005] Ben‐Menahem, D. 2018. “Preparation, Characterization and Application of Long‐Acting FSH Analogs for Assisted Reproduction.” Theriogenology 112: 11–17. 10.1016/j.theriogenology.2017.08.020. 28888334

[rda70216-bib-0006] Bó, G. A. , and R. J. Mapletoft . 2020. “Superstimulation of Ovarian Follicles in Cattle: Gonadotropin Treatment Protocols and FSH Profiles.” Theriogenology 150: 353–359. 10.1016/j.theriogenology.2020.02.001. 32088042

[rda70216-bib-0007] Byambaragchaa, M. , S. H. Park , M.‐H. Park , M.‐H. Kang , and K.‐S. Min . 2025. “Enhanced Production and Functional Characterization of Recombinant Equine Chorionic Gonadotropin (Rec‐eCG) in CHO‐DG44 Cells.” Biomolecules 15, no. 2: 289. 10.3390/biom15020289. 40001592 PMC11853024

[rda70216-bib-0008] Cabeza, O. I. , N. Parra , R. Cerro , et al. 2024. “Development and Characterization of a Novel Variant of Long‐Acting Bovine Follicle‐Stimulating Hormone (brscFSH).” Theriogenology 226: 76–86. 10.1016/j.theriogenology.2024.05.038. 38865791

[rda70216-bib-0009] Camozzato, J. N. B. , G. Maggi , F. P. de Moraes , et al. 2026. “Recombinant Equine Chorionic Gonadotropin for Estrus and Ovulation Induction in Ewes: Effects on Follicular Growth, Luteal Function and Fertility.” Theriogenology 249: 117668. 10.1016/j.theriogenology.2025.117668. 40945248

[rda70216-bib-0010] Carvalho, P. D. , A. H. Souza , L. Cattaneo , et al. 2026. “Effect of Recombinant Equine Chorionic Gonadotropin on Fertility of Lactating Dairy Cows.” Theriogenology 259: 117907. 10.1016/j.theriogenology.2026.117907. 41861708

[rda70216-bib-0011] Cattaneo, L. , C. Prieto , D. Ojeda , A. Pereira , J. Frutos , and G. A. Bó . 2024. “The Use of a Recombinant Equine Chorionic Gonadotropin (reCG) in Fixed‐Time AI Programs in Beef Cattle.” Theriogenology 227: 77–83. 10.1016/j.theriogenology.2024.07.011. 39029411

[rda70216-bib-0012] Cole, H. H. , and G. H. Hart . 1930. “The Potency of Blood Serum of Mares in Progressive Stages of Pregnancy in Effecting the Sexual Maturity of the Immature Rat.” American Journal of Physiology‐Legacy Content 93, no. 1: 57–68. 10.1152/ajplegacy.1930.93.1.57.

[rda70216-bib-0013] Da Silva, S. G. , T. F. Ferreira , J. L. L. A. Correia , et al. 2025. “Recombinant FSH (ZIMBRIA) Improves Blastocyst Yield in Gir Cows Undergoing OPU‐IVF.” Animal Reproduction 22, no. 3: 97.

[rda70216-bib-0014] De Koning, W. J. , G. A. Walsh , A. S. Wrynn , and D. R. Headon . 1994. “Recombinant Reproduction: The Importance of Gonadotrophic and Related Hormones in Veterinary and Human Medicine May Increase With the Adoption of Recombinant Production Methods.” Biotechnology 12, no. 10: 988–992. 10.1038/nbt1094-988. 7765410

[rda70216-bib-0015] De Rensis, F. , and F. López‐Gatius . 2014. “Use of Equine Chorionic Gonadotropin to Control Reproduction of the Dairy Cow: A Review.” Reproduction in Domestic Animals 49, no. 2: 177–182. 10.1111/rda.12268. 24456154

[rda70216-bib-0016] Flores‐Flores, G. , E. Velázquez‐Cantón , M. Boeta , and L. Zarco . 2014. “Luteoprotective Role of Equine Chorionic Gonadotropin (eCG) During Pregnancy in the Mare.” Reproduction in Domestic Animals 49, no. 3: 420–426. 10.1111/rda.12290. 24617452

[rda70216-bib-0017] Frata, M. M. , W. Marques de Lima , M. T. Rovani , et al. 2025. “Single‐Dose Recombinant FSH as a Viable Alternative to Multi‐Dose Porcine FSH in Commercial Superovulation Protocols in Cows.” Theriogenology 244: 117498. 10.1016/j.theriogenology.2025.117498. 40403572

[rda70216-bib-0018] Gifre, L. , A. Arís , À. Bach , and E. Garcia‐Fruitós . 2017. “Trends in Recombinant Protein Use in Animal Production.” Microbial Cell Factories 16: 40. 10.1186/s12934-017-0654-4. 28259156 PMC5336677

[rda70216-bib-0019] Gretter, A. V. , L. M. Rebeis , C. M. Martins , et al. 2025. “In Vivo Embryo Production Using Recombinant Hormones (rFSH and reCG) for Superovulation in Nelore ( *Bos indicus* ) Donors.” Animal Reproduction 22, no. 3: 66.

[rda70216-bib-0020] Gutierrez‐Reinoso, M. A. , E. H. Escribano , I. Cabezas , et al. 2025. “Superovulation of Dairy Cows Using Recombinant FSH (bscrFSH): Effect of the Number of FSH Applications on Ovarian Response, Hormone Profiles, and In Vivo Embryo Production.” Theriogenology 234: 42–50. 10.1016/j.theriogenology.2024.12.002. 39644521

[rda70216-bib-0021] Hassanein, E. M. , Z. Szelényi , and O. Szenci . 2024. “Gonadotropin‐Releasing Hormone (GnRH) and Its Agonists in Bovine Reproduction I: Structure, Biosynthesis, Physiological Effects, and Its Role in Estrous Synchronization.” Animals 14, no. 10: 1473. 10.3390/ani14101473. 38791690 PMC11117390

[rda70216-bib-0022] Khodadadi, A. , A. Niasari‐Naslaji , D. Nikjou , and B. Mohammadi . 2022. “Superovulation of High‐Producing Holstein Lactating Dairy Cows With Human Recombinant FSH and hMG.” Theriogenology 191: 239–244. 10.1016/j.theriogenology.2022.08.010. 35998407

[rda70216-bib-0023] Laster, D. B. 1972. “Disappearance and Uptake of [125I]FSH in the Rat, Rabbit, Ewe and Cow.” Reproduction 30, no. 3: 407–415. 10.1530/jrf.0.0300407. 4672488

[rda70216-bib-0024] Li, Z.‐M. , Z.‐L. Fan , X.‐Y. Wang , and T.‐Y. Wang . 2022. “Factors Affecting the Expression of Recombinant Protein and Improvement Strategies in Chinese Hamster Ovary Cells.” Frontiers in Bioengineering and Biotechnology 10: 880155. 10.3389/fbioe.2022.880155. 35860329 PMC9289362

[rda70216-bib-0025] Lösle, M. , C. W. Lin , J. Beil‐Wagner , M. Aebi , and T. Buch . 2025. “Comparison of Pregnant Mare Serum Gonadotropin Products With Surprising Differences in Protein Content.” Scientific Reports 15, no. 1: 6824. 10.1038/s41598-025-90833-3. 40000800 PMC11861321

[rda70216-bib-0026] Mirzaei, A. , M. C. Londoño‐Mendez , S. Lasso‐Ramirez , et al. 2024. “Embryo Production by Holstein Heifers Superovulated With a Recombinant Long‐Acting Follicle‐Stimulating Hormone Analog.” Journal of Animal Science 102: skae326. 10.1093/jas/skae326. 39447034 PMC11582645

[rda70216-bib-0027] Murphy, B. D. 2012. “Equine Chorionic Gonadotropin: An Enigmatic but Essential Tool.” Animal Reproduction 9, no. 3: 223–230.

[rda70216-bib-0029] Perecin, F. , A. C. S. Oliveira , H. F. R. Saraiva , et al. 2025. “Effect of Treatment With Recombinant Bovine Somatotropin and Recombinant Follicle Stimulating Hormone on Oocyte Quality of Heifer and Cow Nelore ( *Bos indicus* ) Donors.” Animal Reproduction 22, no. 3: 81.

[rda70216-bib-0030] Rodrigues, A. , A. V. Gretter , A. H. Souza , et al. 2025. “Efficacy of Recombinant Equine Chorionic Gonadotropin in Fixed‐Time Embryo Transfer Protocols for *Bos indicus* and *Bos taurus* Recipients.” Animal Reproduction 22, no. 3: 61.

[rda70216-bib-0031] Rodríguez, A. M. , L. Gelid , M. G. Bilbao , et al. 2024. “Effect of an Equine Chorionic Gonadotrophin‐Like Recombinant Glycoprotein Treatment on Fertility in Angus Cattle.” Theriogenology 227: 84–91. 10.1016/j.theriogenology.2024.07.013. 39032226

[rda70216-bib-0032] Stewart, F. , and W. R. Allen . 1981. “Biological Functions and Receptor Binding Activities of Equine Chorionic Gonadotrophins.” Reproduction 62, no. 2: 527–536. 10.1530/jrf.0.0620527. 6265633

[rda70216-bib-0033] Viana, J. H. M. , R. M. de Moura , L. P. Martins , R. A. Figueiredo , L. G. B. Siqueira , and C. A. C. Fernandes . 2024. “Superovulating Cattle With Corifollitropin‐Alpha, a Long‐Acting Recombinant Human FSH (rhFSH): Dose‐Response, Half‐Life, Effects on the Ovaries, and Embryo Outcomes.” Theriogenology 226: 302–307. 10.1016/j.theriogenology.2024.06.033. 38959840

[rda70216-bib-0034] Vilanova, X. M. , N. De Briyne , B. Beaver , and P. V. Turner . 2019. “Horse Welfare During Equine Chorionic Gonadotropin (eCG) Production.” Animals 9, no. 12: 1053. 10.3390/ani9121053. 31805698 PMC6940776

[rda70216-bib-0035] Villarraza, C. J. , S. Antuña , M. B. Tardivo , et al. 2021. “Development of a Suitable Manufacturing Process for Production of a Bioactive Recombinant Equine Chorionic Gonadotropin (reCG) in CHO‐K1 Cells.” Theriogenology 172: 8–19. 10.1016/j.theriogenology.2021.05.013. 34082223

[rda70216-bib-0036] Villarraza, J. , S. Antuña , M. B. Tardivo , et al. 2024. “Development of a Biotechnology Process for the Production of a Novel Hyperglycosylated Long‐Acting Recombinant Bovine Follicle‐Stimulating Hormone.” Biotechnology Journal 19, no. 6: e2400260. 10.1002/biot.202400260. 38900054

